# A Gold Nanoparticle-based Aptasensor for Specific Detection of CA125

**DOI:** 10.31661/gmj.v13i.3180

**Published:** 2024-01-17

**Authors:** Ghasem Ebrahimi, Parvin Samadi Pakchin, Maryam Mousivand, Amirabbas Jalili Bolhasani, Ali Mota

**Affiliations:** ^1^ Research Center for Pharmaceutical Nanotechnology, Biomedicine Institute, Tabriz University of Medical Sciences, Tabriz, Iran; ^2^ Department of Clinical Biochemistry and Laboratory Medicine, Faculty of Medicine, Tabriz University of Medical Sciences, Tabriz, Iran; ^3^ Microbial Biotechnology Department, Agricultural Biotechnology Research Institute of Iran, Agricultural Research, Education and Extension Organization, Karaj, Iran

**Keywords:** Aptamer, Label-free Aptasensor, AuNP Aggregation, Ovarian Cancer, CA-125 Antigen

## Abstract

Background: In this work, an aptamer-based biosensor was successfully developed based on the salt-induced gold nanoparticle (AuNP) aggregation phenomenon for the detection of carbohydrate antigen 125 (CA125), which is an important tumor marker for ovarian cancer. Materials and Methods: Citrate-coated AuNPs are relatively highly dispersed NPs. In the presence of different salts, the electrostatic stability of NPs is reduced, and depending on the type of salt and its concentration, different degrees of aggregation occur. On the other hand, the aptamer is easily adsorbed on the AuNP surface and can prevent salt-induced AuNP aggregation. This phenomenon was used in this study to develop a simple biosensor for the detection of CA125. Results: In the presence of CA125, the aptamer was desorbed from the AuNP surface to bind to its antigen due to the higher affinity, leading to the aggregation of AuNPs and a change in the absorption spectra of the solution. Under the optimum condition, the fabricated aptasensor showed a linear range of 15-160 U/mL with a limit of detection (LOD) of 14.41 U/mL. Conclusion: The aptasensor exhibited good repeatability with notable selectivity with regard to CA125 detection, even in human serum samples, as compared to the enzyme-linked immunosorbent assay (ELISA). In conclusion, the engineered aptasensor can serve as a promising tool for the simple, rapid, and cost-effective detection of CA125.

## Introduction

Ovarian cancer is a gynecologic cancer, and because of the lack of specific symptoms in the early stage, it has the highest mortality rate among women worldwide [1][2]. A crucial tool for the early diagnosis and monitoring of cancer is the detection of tumor markers [3][4]. Carbohydrate antigen 125 (CA125) is a tumor marker that holds great significance for the early diagnosis of ovarian cancer [5]. CA125 is a mucin-like transmembrane glycoprotein that is a component of respiratory, ocular, and female reproductive tract epithelia [6][7] and was first introduced as a tumor marker by Bast et al. in 1983 [8]. Its blood levels are elevated in around 90% of women with advanced ovarian cancer, which makes it an effective indicator for the diagnosis, treatment, and monitoring of epithelial ovarian cancer [9]. Moreover, CA125 has been utilized as a biomarker for breast, gastric, lung, and liver cancers [10].

Currently, different techniques are employed for CA125 detection, including the enzyme-linked immunosorbent assay (ELISA) [11][12], chemiluminescence assays [13], chemiluminescence-capillary electrophoresis (CL-CE) [14], microchip electrophoresis [15], electrochemiluminescent immunoassay [16], resonance Rayleigh scattering (RRS) [17], fluorescence resonance energy transfer analysis [18], and electrochemical assays [19][20], among others. Although these methods are well known and offer several advantages, they still have certain drawbacks, such as high cost, unstable substrates, complex procedures, large sample sizes, and long detection times, which potentially affect the detection efficiency and practicality. Thus, developing new methods with simple procedures, low cost, and high efficiency for the detection of CA125 has become a challenging and significant endeavor.

Recently, some aptamer-based biosensors (aptasensors) using gold nanoparticles (AuNPs) have been reported for the simple and low-cost detection of biomarkers [21][22][23][24]. AuNPs have drawn much attention for the development of nanobiosensors for novel bioassays owing to their distinct chemical and physical characteristics and size/distance-dependent optical properties, as well as their simple and efficient mechanisms [25][26][27]. Under normal conditions, citrate-coated AuNPs are well dispersed in water because of the electrostatic repulsion of the citrate negative charge, with a lmax around 520 nm. When AuNPs aggregate (by adding a salt) and turn into larger nanoparticles, lmax shifts to a larger wavelength. On the other hand, the surface adsorption of an oligonucleotide (such as an aptamer) on the surface of AuNPs prevents the salt-induced aggregation of AuNPs by establishing steric stability [23][28]. An aptamer is a chemically synthesized single-stranded DNA (ssDNA) or RNA molecule that is selected by systematic evolution of ligands through the exponential enrichment (SELEX) process and can be an alternative to other natural reporters, such as antibodies and enzymes. Aptamers can bind to a broad range of target molecules, exhibit satisfactory stability, are easy to modify, and can be produced on a large scale [29][30]. Hence, they have served as potent bio-sensing molecules for the biorecognition of biomolecules and in conjunction with AuNPs can be applied as a powerful sensing method for biomarker detection.

In this study, we designed a label-free strategy for specific and simple detection of CA125 using unmodified AuNPs as probes and the ssDNA aptamer as a recognition element. The concentrations of the salt and aptamer were also successfully optimized in order to improve detection sensitivity. Furthermore, the application of this assay for the direct detection of CA125 in human serum samples was investigated.

## Materials and Methods

Materials and Reagents

Chloroauric acid trihydrate (HAuCl4·3H2O), trisodium citrate (C6H5Na3O7), acetic acid, and sodium chloride (NaCl) were obtained from Sigma-Aldrich Corporation (St. Louis, USA). ssDNA (5’-TCA CTA TAG GGA GAC AAG AAT AAA CGC TCA A- 3’) [9] was synthesized by TAG Copenhagen A/S (Denmark) at a synthesis scale of 0.2 mmol. The CA125 antigen was purchased from Fujirebio (Göteborg, Sweden). Monopotassium phosphate and potassium chloride were purchased from Merck Group (Darmstadt, Germany).

Instrumentation or Apparatus

The UV-Vis absorption spectra were recorded on the Cytation™ 5 Cell Imaging Reader (BioTek, Winooski, USA). The Milli-Q system at 18 MU was utilized for water purification throughout the experiments.

Synthesis of AuNPs

Citrate-coated AuNPs were synthesized via the reduction of HAuCl4 with trisodium citrate and characterized by UV-Vis spectrometry [21]. In brief, the HAuCl4 solution (1 mM) was heated to a boiling point while being stirred. Upon boiling, the trisodium citrate solution (38.8 mM) was quickly added, and the mixture was boiled for another 20 min under stirring. Eventually, the color of the solution gently altered to purple-red, indicating AuNP formation. It was then allowed to cool to room temperature under constant stirring and stored at 4ºC.

Aptasensor Preparation

First, 50 µL of CA125 at different concentrations (15, 20, 30, 40, 60, 80, 120, and 160 U/mL) was incubated with 50 µL of the aptamer (200 nM) for 30 min at room temperature in microplate wells. Then 50 µL of AuNPs (~13 nm) was added to each microplate well and allowed to equilibrate for 5 min. After adding 10 µL of NaCl (1 M) and incubating for 5 min, the optical density (OD) shift was measured using a microplate reader with filters at 660 and 520 nm. The concentrations of the salt and aptamers, which are the two key parameters, were optimized in order to improve the sensitivity of detection. Moreover, the selectivity and repeatability of the designed aptasensor were evaluated. In addition, the standard addition recovery method was utilized to evaluate the aptasensor applicability in the human serum sample.

Molecular Docking Analysis

Molecular docking was performed to investigate the binding mode between the aptamer and CA125 using the Patchdock online server [31]. For the docking simulation, the three-dimensional (3D) structures of CA125 and the aptamer were realized. The 3D structures of CA125 were drawn by Iterative Threading ASSEmbly Refinement (I-TASSER) [32]. The 3D structure of the aptamer sequence was predicted by a pipeline previously reported by Mousivand et al. [33]. All possible binding modes between the aptamer and CA125 were assessed in the rotational and translational space by Patchdock as an automated tool, and an energy-based scoring function was used to evaluate each pose [31]. Finally, the most optimal docking pose was selected. The graphical illustration of aptamer-CA125 complexes and their interactions was generated by Molecular Operating Environment (MOE) v2019.0102 x64 and BIOVIA Discovery Studio v21.1.0.20298.

## Results

**Figure-1:**
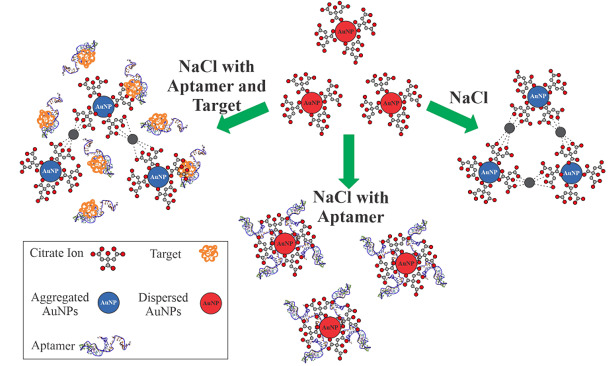
Figure 1. Schematic illustration of the aptamer-based sensor for CA125 detection based on the salt-induced AuNP aggregation phenomenon. The citrate-coated AuNPs are well dispersed in water. In the presence of NaCl, the negative charge of the citrate ion is neutralized, which induces the aggregation of AuNPs. The aptamer can adsorb on the surface of AuNPs and resulting in their stability against aggregation. In the presence of the CA125, the aptamer could no longer be adsorbed onto the surface of the AuNPs to prevent them from aggregation.

**Figure-2:**
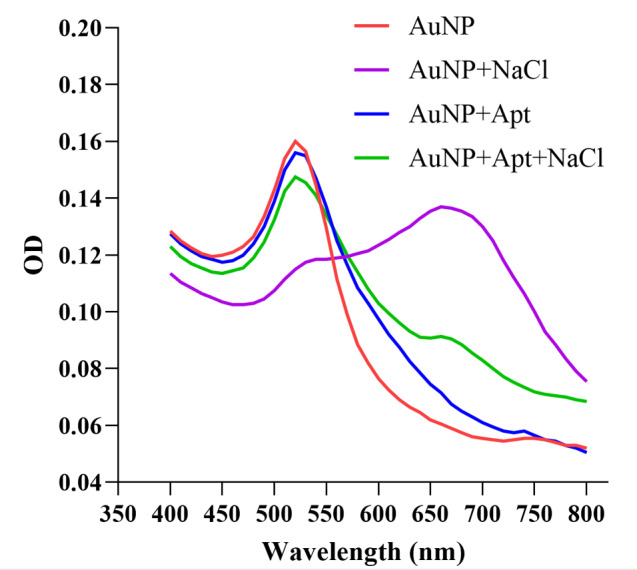
Figure 2. Absorption spectra of the AuNP solution under various conditions. The NPs have the maximum absorbance at 520 nm (red trace). In the presence of NaCl, AuNPs aggregate and a new peak at around 660 nm appears (purple trace). The aptamer does not affect the absorption spectra of the AuNP solution (blue trace) whereas it prevents NaCl-induced aggregation (green trace).

**Figure-3:**
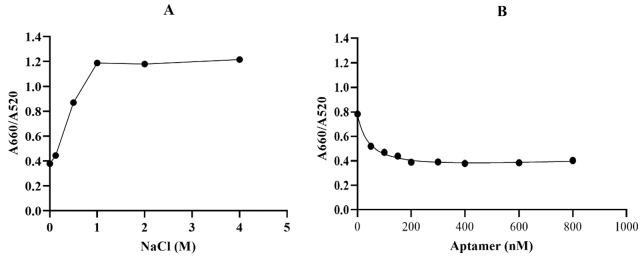
Figure 3. Optimization of the key parameters of the label-free aptasensor. The A660/A520 ratio of AuNPs with A) different concentrations of NaCl and B) different concentrations of the CA125 aptame

**Figure-4:**
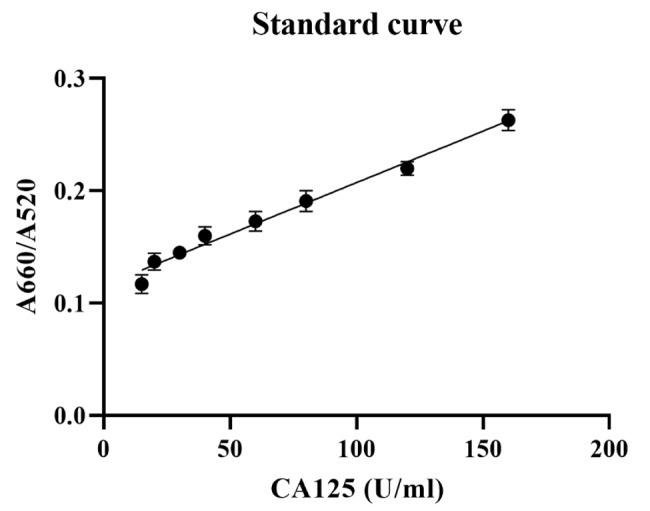
Figure 4. A660/A520 plotted against the CA125 concentration under optimized experimental conditions.

**Figure-5:**
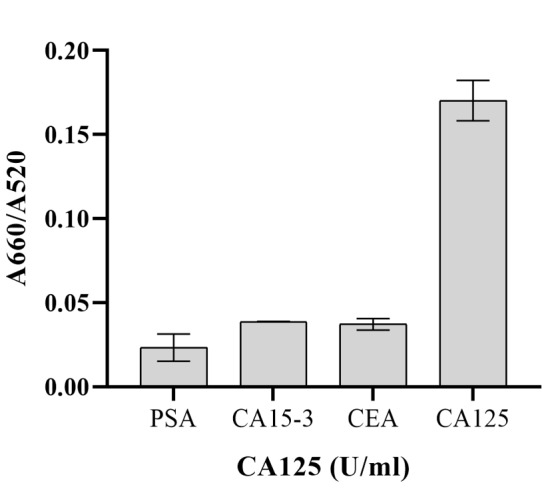
Figure 5. Selectivity of the designed aptasensor for CA125. The results show that the A660/A520 ratio was very low for PSA (50 ng/mL), CA15-3 (50 U/mL), and CEA (50 ng/mL), while the aptasensor displayed an excellent sensitivity toward 50 U/mL CA125.

**Figure-6:**
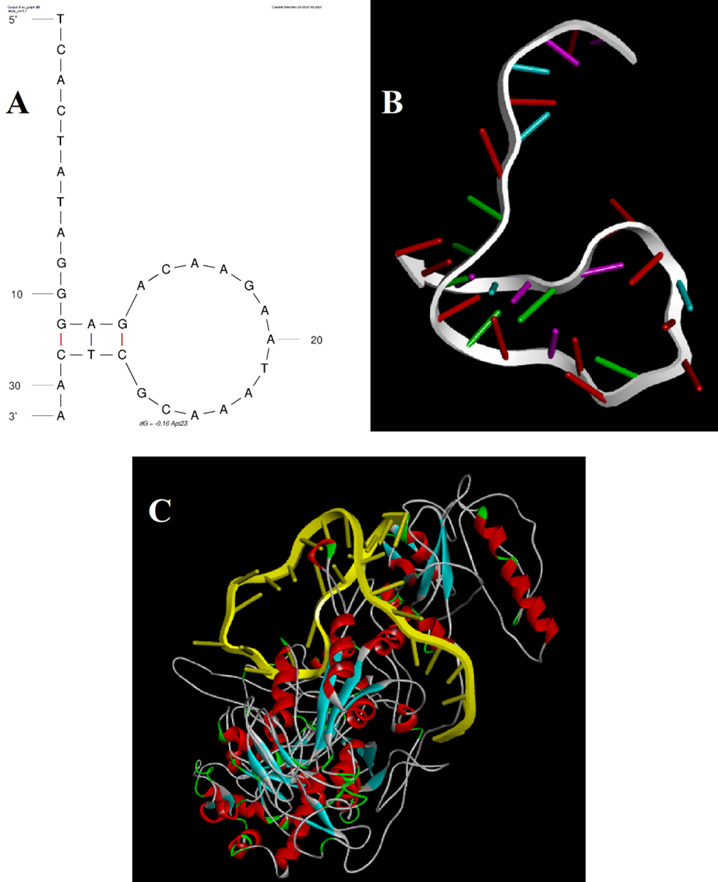
Figure 6. Molecular docking analysis. A) The 2D secondary structure of the 31-mer aptamer with a typical stem-loop structure. B) The 3D aptamer structure predicted by a pipeline previously reported by Mousivand et al. [33]. C) The binding mode between the aptamer and CA125 demonstrated by molecular docking using the Patchdock online server.

The UV-visible results of the designed AuNP aptasensor under different experimental conditions are shown in Figure-2. The dispersed and aggregated AuNPs displayed λmax of around 520 and 660 nm, respectively. As demonstrated in Figure-3A and 3B, the NaCl concentration of 1 M was selected as the optimal salt concentration, and 200 nM was chosen as the optimal aptamer concentration for this experiment. The sensor response was assessed using the A660/A520 ratio plotted against the CA125 concentration (Figure-4). The A660/A520 ratio showed a good linearity between 15 and 160 U/mL with a limit of detection (LOD) of 14.41 U/mL. Also, in presence of some common interferences, the selectivity results demonstrated that a significant rise in the A660/A520 ratio was obtained only for CA125 (Figure-5). As shown in Table-2, the recovery rate and RSD values detected by the aptasensor range from 76% to 107% and 1.85 to 7.40, respectively. In addition, the results of the proposed aptasensor were in good agreement with those of the ELISA method (Table-3). The predicted two-dimensional (2D) aptamer displayed a typical stem-loop structure (Figure-6A). The docking score of CA125 with the aptamer, as evaluated by Patchdock, was 21752. Moreover, the binding pocket is located in the SEA12, SEA13, and SEA15 domains of CA125, and consisted of hydrogen, hydrophobic, and electrostatic bonds.

## Discussion

**Table:** Table1. Evaluation of the Aptasensor’s Repeatability

	1	2	3	4	5	6	RSD
A660/A520	0.360	0.356	0.353	0.342	0.356	0.377	3.18

**(RSD: relative standard deviation)**

**Table:** Table2. Estimation of CA125 Recoveries in a Diluted Serum Sample

Added CA125 concentration (U/mL)	Average value of measured concentration of CA125 (U/mL) N=10	Recovery (%)	RSD
15	11.38	76	4.72
20	19.73	99	1.85
30	30.51	102	2.76
40	42.72	107	6.33
60	56.96	95	3.61
80	81.37	102	5.24
120	113.45	95	3.24
160	155.13	97	7.40

**Table:** Table3. A Comparison between the ELISA Method and the Proposed Aptasensor

Sample ID	ELISA (U/ml)	Aptasensor (U/ml)
1	111	113.59
2	138	133.22
3	148	148.77
4	32.7	28.81
5	35.7	36.50

Principle of the Assay

The idea of using unmodified AuNPs and DNA probes to detect CA125 originated from the finding that citrate-coated AuNPs aggregate in the presence of high concentrations of salt, such as a NaCl solution [28][33]. The AuNPs are stabilized by the negatively charged citrate ions because their electrostatic repulsion inhibits the AuNPs from aggregating. The citrate-coated AuNPs are well dispersed in water. In the presence of NaCl, the negative charge of the citrate ion is neutralized, which induces the aggregation of AuNPs. Thus, the intensity of their lmax reduces and a new peak at around 660 nm emerges. A random coil ssDNA (here the CA125 aptamer) can adsorb on the surface of AuNPs via electrostatic interactions between AuNPs and the bases of ssDNA and add extra negative charges and steric stability to AuNPs, resulting in their stability against NaCl-induced aggregation. Due to the high affinity of the aptamer for its target, in the presence of the target (here CA125), the conformation of ssDNA switches from the random coil structure to a rigid secondary structure, allowing it to interact with the target. Therefore, the aptamer could no longer be adsorbed onto the surface of the AuNPs to prevent them from NaCl-induced aggregation, thus resulting in an increase in A660/A520, which has a linear positive correlation with the CA125 concentration within a certain range. The mechanism of the CA125 biosensor is illustrated in Figure-1. According to this rationale, an optical aptasensor was developed for the detection of CA125 based on salt-induced AuNP aggregation.

The UV-visible results of the designed AuNP aptasensor under different experimental conditions are shown in Figure-2. The dispersed AuNPs show a lmax of around 520 nm. In the presence of NaCl, the NPs aggregate and a new peak at around 660 nm appears. The adsorption of the aptamer onto the AuNPs does not affect the absorption spectra of AuNPs while precluding the NaCl-induced aggregation.

Optimization of the Key Parameters

The concentration of the salt and aptamers, which are the two key parameters, should be optimized in order to improve detection sensitivity. Optimization of reaction conditions leads to the development of a sensitive aptasensor for the detection of CA125. In the first step, AuNPs were mixed with different concentrations of NaCl (0, 0.125, 0.25, 0.5, 1, 2, and 4 M). As shown in Figure-3A, the aggregation of the mixture started at a NaCl concentration of 0.2 M, and 1 M was the minimum NaCl concentration that could induce complete AuNP aggregation, resulting in the maximum A660/A520 ratio. Therefore, the NaCl concentration of 1 M was selected as the optimal salt concentration. In order to optimize the CA125 aptamer concentration, different concentrations of the aptamer (0, 50, 100, 150, 200, 300, 400, 600, and 800 nM) were incubated with 50 µL of AuNPs (~13 nm) for 5 min, followed by the addition of the optimized NaCl concentration. As indicated in Figure-3B, 200 nM is the maximum aptamer concentration that can prevent salt-induced AuNP aggregation. Based on this finding, 200 nM was chosen as the optimal aptamer concentration for this experiment. Moreover, this result implies that the CA125 aptamer is efficiently adsorbed onto the AuNPs and stabilizes them in the presence of NaCl.

Analytical Performance of the Aptasensor

Figure-4 displays the UV-Vis results of the developed aptasensor for CA125 detection. The sensor response was assessed using the A660/A520 ratio plotted against the CA125 concentration. The A660/A520 ratio showed a good linearity between 15 and 160 U/mL with a limit of detection (LOD) of 14.41 U/mL. The LOD was calculated using the formula 3.3(a/slope), where "a" represents the standard deviation and the slope is obtained from the linear calibration plot. The correlation coefficient (R2) of the standard curve was 0.9831.The aptasensor’s selectivity is one of the crucial features for analyzing biological samples without the separation steps.

The selectivity of this aptasensor for CA125 was evaluated via determining the A660/A520 ratios of some common interferences, such as carcinoma antigen 15-3 (CA15-3), prostate-specific antigen (PSA), and carcinoembryonic antigen (CEA), under the same conditions and comparing them with the response of the designed aptasensor to CA125. The results demonstrated that a significant rise in the A660/A520 ratio was obtained only for CA125 (Figure-5).

To evaluate the method’s precision, repeatability was calculated. The relative standard deviation (RSD) value of the four measurements was 3.18%, indicating that the aptasensor’s repeatability was acceptable (Table-1).

Aptasensor Application in the Analysis of Serum Samples

To evaluate the applicability of the aptasensor for CA125 sensing in human serum samples, the standard addition recovery method was performed in a 10-fold diluted human serum sample. As shown in Table-2, the recovery rate and RSD values detected by the aptasensor range from 76% to 107% and 1.85 to 7.40, respectively. In addition, for more verification, the results obtained from the designed aptasensor were compared with those of the ELISA method in five human serum samples. It was observed that the results of the proposed aptasensor were in good agreement with those of the ELISA method (Table-3). Finally, the cutoff level of CA125 is 35 U/mL and the developed aptasensor can be utilized for clinical applications with good linear range, reliability, and accuracy.

Molecular Docking Analysis

Molecular docking methods have been employed to identify possible bonding mechanisms between CA125 and the aptamer (Figure-6). The predicted two-dimensional (2D) aptamer displayed a typical stem-loop structure (Figure-6A). As previously indicated, aptamer stem-loop structures play a pivotal role in ligand and receptor binding [34][35][36]. The docking score of CA125 with the aptamer, as evaluated by Patchdock, was 21752, which indicates efficient binding. The binding pocket of the aptamer and CA125 consisted of hydrogen, hydrophobic, and electrostatic bonds. Moreover, the binding pocket is located in the SEA12, SEA13, and SEA15 domains of CA125, which always exist in the extracellular region and function in diverse recognition events [37].

## Conclusion

In this study, a spectrophotometric aptasensor based on a DNA aptamer and unmodified AuNP aggregation was successfully fabricated for the detection of CA125. The linear dynamic range and LOD were found to be 15-160 U/mL and 14.41 U/mL of CA125 with good repeatability, which is of great importance in cancer biomarker detection. This wide linear detecting range was successfully attained by optimizing the two key parameters, i.e., the concentration of the salt and aptamers, in order to control the aggregation of AuNPs. This method demonstrated good selectivity against CA125 and avoided disruptions from common interferences, such as CA15-3, PSA, and CEA. Furthermore, the aptasensor was effectively used in CA125 detection in serum samples with a recovery rate of 76% to 107%. The developed aptasensor is inexpensive, easy to use, does not need a trained user, and can be applied in the complex matrix of the serum. Therefore, it can be regarded as a promising tool in clinical applications.

## Acknowledgments

The authors would like to thank the Research Center for Pharmaceutical Nanotechnology at Tabriz University of Medical Sciences for supporting this project as part of a Ph.D. thesis (#63766).

## Conflict of Interest

The authors declare that they have no known competing financial interests or personal relationships that could have appeared to influence the work reported in this paper.
